# Acoustic Microfluidic Separation Techniques and Bioapplications: A Review

**DOI:** 10.3390/mi11100921

**Published:** 2020-10-02

**Authors:** Yuan Gao, Mengren Wu, Yang Lin, Jie Xu

**Affiliations:** 1 Department of Mechanical and Industrial Engineering, University of Illinois at Chicago, Chicago, IL 60607, USA; mwu50@uic.edu (M.W.); jiexu@uic.edu (J.X.); 2 Department of Mechanical, Industrial and Systems Engineering, University of Rhode Island, Kingston, RI 02881, USA; yanglin@uri.edu

**Keywords:** microfluidics, acoustic separation techniques, bioapplications, BAW, SAW

## Abstract

Microfluidic separation technology has garnered significant attention over the past decade where particles are being separated at a micro/nanoscale in a rapid, low-cost, and simple manner. Amongst a myriad of separation technologies that have emerged thus far, acoustic microfluidic separation techniques are extremely apt to applications involving biological samples attributed to various advantages, including high controllability, biocompatibility, and non-invasive, label-free features. With that being said, downsides such as low throughput and dependence on external equipment still impede successful commercialization from laboratory-based prototypes. Here, we present a comprehensive review of recent advances in acoustic microfluidic separation techniques, along with exemplary applications. Specifically, an inclusive overview of fundamental theory and background is presented, then two sets of mechanisms underlying acoustic separation, bulk acoustic wave and surface acoustic wave, are introduced and discussed. Upon these summaries, we present a variety of applications based on acoustic separation. The primary focus is given to those associated with biological samples such as blood cells, cancer cells, proteins, bacteria, viruses, and DNA/RNA. Finally, we highlight the benefits and challenges behind burgeoning developments in the field and discuss the future perspectives and an outlook towards robust, integrated, and commercialized devices based on acoustic microfluidic separation.

## 1. Introduction

The successful separation of target substances from solutions is a key step of significant importance in a variety of applications, such as biomedical applications, biochemical detection and analysis, food processing, and water treatment [1,2,3,4]. As an old but pivotal process, numerous approaches have been developed and widely applied to separation, including centrifugation, electrophoresis, size-based filtration, solid-phase extraction, and solvent addition [5,6,7,8,9]. Despite great performance achieved, a majority of traditional approaches used in research and clinical applications require complex preparation, bulky instruments, and high sample volume, which do not align with today’s research trends: low energy consumption, short reaction time, low cost, high portability, and low minimum volume requirement. Therefore, microfluidic separation has become a promising alternative that meets these requirements.

The ability to convert a mixture of biological samples into distinct populations is critical in many applications. For instance, isolating circulating tumor cells (CTCs) in the early stage of cancer can be applied for examining the efficiency of personalized cancer treatment [10]. By separating white blood cells (WBCs) from blood, heart disease [11,12], pneumonia [12], and HIV [13] can be diagnosed. The separation of sperm and epithelial cells has the potential to impact forensic DNA analysis of sexual assault evidence [14]. To achieve these separation goals, microfluidic separation also has the advantage of integrating into various excitation systems, including acoustic, electrical, magnetic, optical, and mechanical systems [9,15,16,17,18]. Specifically, acoustic separation techniques that utilize the acoustic field can separate particles depending on their mechanical properties. By applying a nonuniform electric field, dielectrophoresis (DEP) force can be generated and used to separate particles and cells based on their dielectric properties. Magnetic separation techniques can sort cells or particles by magnetic force. For optical separation, particles can be separated by the change of momentum of photon-produced force. Mechanical separation, such as pinched flow fractionation (PFF), inertia and dean flow fractionation, filtration, and deterministic lateral displacement (DLD), can separate particles with the use of interaction between the particles, the flow field, and microchannel structure [19]. Among the various techniques, acoustic microfluidic separation technology emerges with a combination of strengths that is suitable to separate biological samples effectively: label-free, contactless, and biocompatible. Without contact, this technology is able to separate samples based on their different physical properties, such as density, size, and compressibility [18,20,21]. Additionally, the applied acoustic field is versatile and highly controllable. Furthermore, by properly designing the device and controlling the operation conditions, the biocompatibility and cell viability have been examined, which demonstrated that the viability and characteristics of the biological samples are almost not damaged under the proper frequency range [22,23].

Although several excellent review articles [19,24,25] have reported relevant acoustic microfluidic separation techniques and applications, a comprehensive and thorough overview of fundamental theory and mechanisms that underpin these achievements was not reported. This review aims to bridge the gap between separation phenomena and underlying physics for a wide range of audiences, especially those who are new to the field. In addition, we also highlight the biological applications of these techniques and discuss the limitations and perspectives of the acoustic microfluidic separation.

## 2. Theory and Background

In this section, we will briefly introduce the type of acoustic waves, the wave propagation inside the microchannel, and their fundamental mechanisms in microfluidic separation.

### 2.1. Acoustic Wave

**Figure 1:**
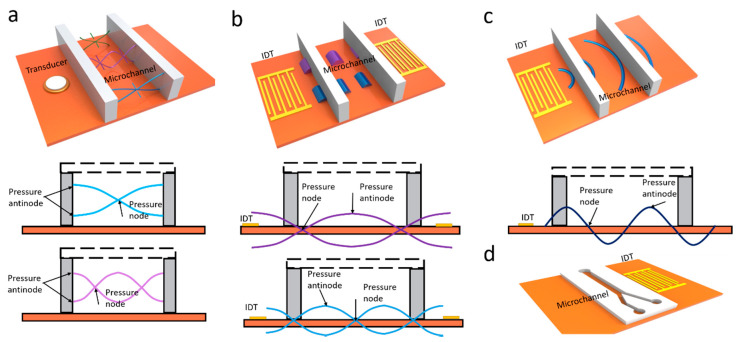
(**a**) Schematic illustration of propagation of bulk acoustic waves (BAWs) in a microchannel; (**b**) Schematic illustration of propagation of standing surface acoustic waves (SSAWs) in a microchannel; (**c**) Schematic illustration of propagation of traveling surface acoustic waves (TSAWs) in a microchannel; (**d**) Schematic illustration of an acoustic microfluidic device. IDT—interdigitated transducer.

An acoustic wave is a type of mechanical wave that propagates along a longitudinal wave driven by an acoustic source, generated by the mechanical stress from a piezoelectric transducer. Acoustic waves can be categorized into two types: surface acoustic waves (SAWs) and bulk acoustic waves (BAWs). Both have been widely used to manipulate micro objects in the field of microfluidics [26]. SAWs were first explored by Lord Rayleigh in 1885, also known as Reynolds SAWs, which is widely applied in microfluidics [27]. There are two types of SAW-driven microfluidics: traveling SAWs (TSAWs) and standing SAWs (SSAWs). Traveling surface acoustic waves (TSAWs) are defined as the SAWs that propagate in one direction and radiate away from the acoustic sources (Figure 1c). Standing surface acoustic waves (SSAWs) are generated by two opposite traveling SAWs interfering or a reflecting traveling SAWs, creating fixed nodes and antinodes in an open or confined domain (Figure 1b) [28].

Conversely, bulk acoustic waves (BAWs) are standing waves that propagate inside the resonant chamber of the microchannel. In BAW-based microfluidic devices, the piezoelectric transducer is bonded on the microchannels and actuated by an AC power supply to generate BAWs. Unlike SAWs, which spread along the surface of the material, bulk acoustic waves propagate inside the bulk of the material (Figure 1a). For this reason, BAW-based microfluidic devices generally require more energy than SAW-based microfluidic devices in order to achieve similar acoustic effects [29,30].

To understand the mechanism of acoustic microfluidic separation, it is important to define the forces that the particles may experience in the microchannel when they are exposed to acoustic fields. Generally, with acoustic actuation, microparticles suspended in fluids may experience both drag force and acoustic radiation force.

### 2.2. Drag Force

Streaming flow, a steady flow in a fluid, divided into acoustic streaming and microstreaming, has been widely studied in acoustic microfluidic separation [31,32,33]. Acoustic streaming is created when high-amplitude acoustic oscillations generate gradients when the acoustic energy is attenuated in the fluid due to fluid viscosity. The microstreaming phenomenon is induced by the oscillations of a small object such as a microbubble in the fluid as the local gradients are high on a small scale [32]. Both types of streaming flow will induce the Stokes drag force, which can dominate the suspended particles around the streaming flow. At a low Reynolds number, the Stokes drag force acted on a particle can be calculated by an equation given below:

(1) $$F_{D}=\;-6\pi \mu R_{p}v$$

where μ denotes the fluid viscosity, v is the relative velocity between the fluid and particles, $R_{p}$ is the radius of the particle.

### 2.3. Acoustic Radiation Force

Acoustic waves propagating through the liquid generate a pressure gradient that produce the acoustic radiation force (ARF) in the acoustic field. There are two types of acoustic radiation force: the primary radiation force (PRF) and the secondary radiation force (SRF). The expression of primary radiation force in a traveling wave is derived by King [34], which is given by:

(2) $$F=2\pi \rho_{l}{\left|A\right|}^{2}{\left(kR_{p}\right)}^{6}\frac{9+2{\left(1-\lambda_{\rho }\right)}^{2}}{9{\left(2+\lambda_{\rho }\right)}^{2}}$$

(3) $$\lambda_{\rho }=\frac{\rho_{l}}{\rho_{p}}$$

where A is the complex amplitude of velocity potential; k is the wavenumber of the acoustic radiation, which is equal to 2π/λ; λ is the wavelength;$\;R_{p}$ is the radius of the particle;$\;\rho_{l}$, $\rho_{p}$ is the density of the surrounding fluid and the particle, respectively. Using the equation above, Tan et al. quantitatively estimate the TSAW-based acoustic radiation force between different-sized particles [35]. They found that larger particles experience stronger (two orders of magnitudes) acoustic radiation force than smaller particles at a fixed operating frequency, making the effective separation possible.

The primary acoustic radiation force generated by a standing wave consists of two components, an axial component and a transverse component. The axial acoustic radiation force, $F_{a}$, along with the propagating direction of the acoustic standing wave is responsible for pushing the particle to the node or antinode, which is commonly applied in SSAW-based separation (Equations (4) and (5)) [34,36]:

(4) $$F_{a}=-\left(\frac{\pi p_{0}^{2}V_{p}\beta_{l}}{2\lambda }\right)\phi \left(\beta ,\rho \right)\sin\left(2kz\right)$$

(5) $$\phi \left(\beta ,\rho \right)=\frac{5\rho_{p}-2\rho_{l}}{2\rho_{p}+\rho_{l}}-\frac{\beta_{p}}{\beta_{l}}$$

where ϕ is acoustic contrast factor; $p_{0}$ is the acoustic pressure amplitude; z is the axial distance from a pressure node;$\;V_{p}$,$\;\beta_{p}$ denote the volume and compressibility of particle;$\;\beta_{l}$ is the compressible of the surrounding fluid. It is worth pointing out that the size of the particle has strong influence on the axial radiation force due to particle volume being proportional to the $F_{a}$ from Equation (4). In addition, the compressibility and density of the particle are important factors that dominate the direction of the axial component. It has been proved that when a particle is approaching the pressure node, the axial acoustic radiation force is two orders of magnitude stronger than the transverse radiation force [37]. However, once the particle reaches the position of the pressure node, the axial component vanishes, and the transverse component is no longer negligible. The transverse component ($F_{t}$) can push the particles close to each other in the nodal plane, which directly depends on the gradient of the acoustic energy ($\nabla \langle E_{ac}\rangle$) [38].

(6) $$F_{t}=3d_{p}^{3}\frac{\rho_{p}-\rho_{l}}{2\rho_{p}+\rho_{l}}\nabla \langle E_{ac}\rangle$$

The secondary radiation force exists when multiple particles or bubbles are excited by the same acoustic wave field, which is also called Bjerknes force when acting on gas bubbles [39]. This force becomes important when multiple particles or bubbles are close to each other and it can be either an attractive force or repulsive force [40]. Since bubbles are commonly used in the separation of particles, here, we show the equation of SRF between a bubble and a particle [41]. The secondary radiation force between a bubble and a particle has been introduced in detail by Doinikov [41].

(7) $$F_{SRF}=4\pi \rho_{l}\frac{\rho_{l}-\rho_{p}}{\rho_{l}+2\rho_{p}}\frac{R_{b}{}^{4}R_{p}{}^{3}}{d^{5}}\omega^{2}\xi^{2}$$

From Equation (7), the secondary radiation force depends on the radius and density of the particles ($R_{p}$ and $\rho_{p}$), radius of bubbles ($R_{b}$), density of the fluid $\left(\rho_{l}\right)$, distance between the bubble and particles (d) as well as the frequency (ω) and amplitude of bubble oscillation (ξ). Whether the attractive or repulsive force is induced is determined by the ratio of the fluid and particle densities [42]. Because the force can keep the specific particles oscillating in a certain range of distances around the bubble, it can be used to trap and aggregate specific samples to realize selective separation, which will be discussed in the next section.

## 3. BAW-Based Separation

Bulk acoustic waves have been applied to microfluidic separations with many benefits, such as flexible placement of transducer, simple, and versatile setups. A BAW-based microfluidic device typically operates with bulk acoustic standing waves in a microchannel between two parallel opposite walls. A piezoelectric transducer can generate BAWs in a fluid-filled microchannel and resonance in the channel with acoustically contrasting materials, such as silicon, polydimethylsiloxane (PDMS), and glass. Compared with SAW-based separation devices, BAW-based devices usually work at a lower frequency and a longer wavelength, which allows for handling larger particles [43].

**Figure 2:**
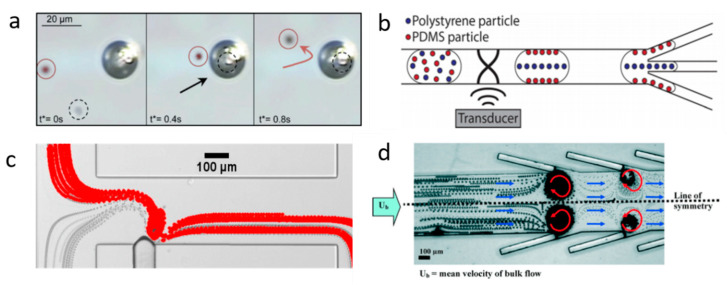
(**a**) At 217 kHz, 5 µm silica particles are trapped by acoustic bubbles, while 5 µm polystyrene particles follow with streamline due to the drag force. Reprinted with permission from reference [52]. (**b**) BAW-based separation of polystyrene and polydimethylsiloxane (PDMS) particles with different acoustic contrast factors. Reprinted with permission from reference [47]. (**c**) Size-sensitive sorting mixture of 5 µm and 2.5 µm particles by acoustic bubble. Reprinted with permission from reference [53]. (**d**) Angled lateral cavity acoustic transducers (LCATs) produce a directional flow from inlet to outlet, large-sized particles are selectively trapped and small-sized particles are transported to the outlet. Reprinted with permission from reference [20].

Using BAW-induced acoustic streaming and acoustic radiation force, Devendran et al. separated particles based on size differences [44]. Actuated by BAW, the acoustic streaming-induced drag force selectively delivered smaller particles to the target location, while the larger particles were dominated by acoustic radiation force. Dauson et al. demonstrated a robust “tilt-angle” BAW-based microfluidic device for the separation of different-sized particles [45]. Tilt angled lead zirconate titanate (PZT) induced primary acoustic radiation force on particles, which deflected particles from the straight path based on the size of the particles. BAW-based devices have also been implemented to separate sub-micron particles from micron particles. Due to the diameter difference, micron particles were dominated by primary acoustic radiation force and focused on the midline of the microchannel, while sub-micron particles were moved toward the sidewall via drag force. Using the same mechanism, they successfully separated bovine red blood cells and *Escherichia coli* (*E. coli*) [46]. In addition to the size-based separation, BAW is also able to separate samples based on density or compressibility. Fornell et al. successfully separated polystyrene and PDMS particles with different acoustic contrast factors inside a water-in-oil droplet (Figure 2b) [47]. Since acoustic radiation forces are proportional to acoustic contrast factors, the polystyrene particles were directed to the pressure nodes while the PDMS particles were moved to the pressure antinodes when BAW is on. By combining with a droplet splitter, these particles were separated in a continuous flow.

### Microbubble-Based Separation

In recent years, acoustically excited bubbles have attracted more and more interest due to their great potential for manipulating objects and fluids in microfluidic applications, which is typically excited by BAWs with relatively low frequencies (kHz) [48,49,50,51]. As mentioned in the theory section, when a bubble is actuated by acoustic waves, the objects near the bubble will experience both secondary radiation force and drag force. Secondary radiation force tends to trap the objects, while microstreaming-induced drag force can transport particles. With ingenious design in force control, many acoustic bubble-based BAW devices have been developed for size or density-based separation for biomedical applications. For example, Rogers et al. demonstrated a density-based method for the separation of same-sized silica beads and polystyrene particles using acoustic bubbles [52]. Due to different densities, the silica beads were trapped by the secondary radiation force, while polystyrene particles, dominated by drag force, were repelled and transported along the streamlines (Figure 2a). This finding shows the acoustic bubble-based microfluidic device has a great potential to be implemented in sorting uniformed and low-concentrated biological samples. Similarly, aided by a syringe pump, size-selective trapping and release were realized via tuning the strength of a Poiseuille flow and a bubble-induced microstreaming, which allows sorting particles of desired size (Figure 2c) [53]. Later, an improved acoustic bubble-based separator termed lateral cavity acoustic transducers (LCATs) is demonstrated, enabling simultaneous self-pumping and size-based cell separation (Figure 2d) [20]. Compared with current bulk lab-based sorting methods, this method is more flexible to integrate with upstream and downstream sample preparation and analysis systems. Our group developed an acoustic bubble array for trapping and releasing a live animal—*Caenorhabditis elegans* (*C. elegans)* [54]. Dominated by secondary radiation force, the *C. elegans* were trapped by the bubble array at a certain frequency and voltage. Gradually decreasing the applied voltage to the actuator, *C. elegans* were released in the order from the biggest to the smallest. This study shows the controllability of the bubble-based device in size-selective trapping and releasing microorganisms. Acoustic bubbles could also be employed in enhancing the separation effect. Zhou et al. integrated an acoustic bubble with pinched flow fractionation (PFF) to enhance particle separation performance, which overcomes the limitation of the conventional PFF method [55]. Xie et al. presented a method for enhancing mass transfer in a liquid–liquid extraction process, which has the potential to be further applied in biochemical separation [56].

## 4. SAW-Based Separation

Interdigitated transducers (IDTs) can be used to generate SAWs. To achieve effective separation, various methods have been explored to control fluids or objects by designing the configuration of IDTs. A single IDT is usually used to generate traveling surface acoustic waves (TSAWs), while two counter IDTs are designed to create standing surface acoustic waves. Both TSAW and SSAW have been applied in acoustic separation, enabling the completion of many difficult separation tasks.

### 4.1. TSAW-Based Separation

TSAW-based separation has been extensively applied in sorting different-sized microparticles, such as polystyrene (PS) particles, fused silica (FS) particles, and polymethyl methacrylate (PMMA) particles. These particles can experience both the acoustic radiation force and the drag force. Whether acoustic streaming flow-induced drag force or acoustic radiation force dominates the moving of the particles in TSAW depends on a dimensionless factor $k_{tr}$ (Equation (8)), which is introduced by Skowronek et al. [57].

(8) $$k_{tr}=2\pi R_{0}/\lambda$$

The experimental results demonstrated that when the perimeter of the particle is smaller than the fluid wavelength (k < 1), the particle would be controlled by the acoustic streaming flow. On the other hand, if the perimeter of the particle is larger than the fluid wavelength (k > 1), the acoustic radiation force would be stronger than the acoustic streaming flow-induced drag force.

**Figure 3:**
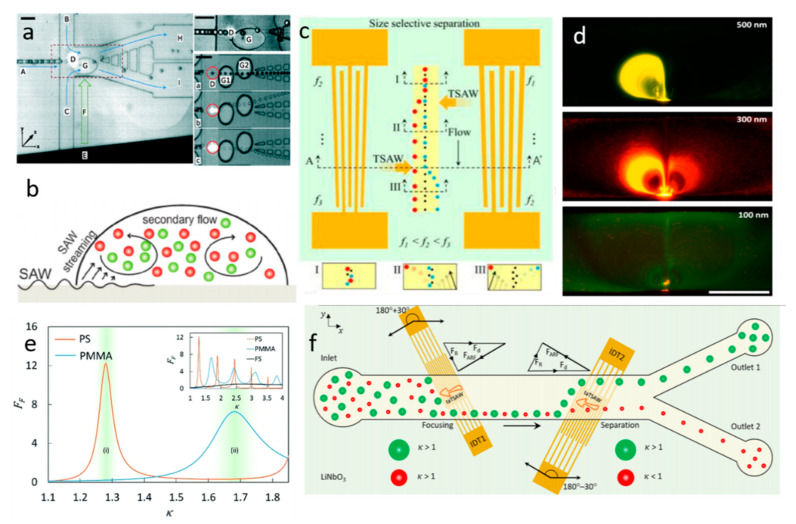
(**a**) TSAW-induced acoustic streaming selectively deflects droplets with fluorescent signal. Reprinted with permission from reference [60]. (**b**) Density-based separation by TSAW-induced acoustic streaming. With TSAW actuation, the lighter particles are distributed at the periphery and more dense particles are accumulated in the center. Reprinted with permission from reference [61]. (**c**) Using two IDTs with different frequencies, mixtures of three sizes of particles are separated by TSAW-induced acoustic radiation force. Reprinted with permission from reference [63]. (**d**) Sub-micron particles separation by TSAW. 500 nm and 300 nm particles are trapped by TSAW-induced radiation force, while 100 nm particles are not trapped due to the stronger drag force. Reprinted with permission from reference [65]. (**e**) Different acoustic radiation force for same-sized particles. Reprinted with permission from reference [64]. (**f**) Tilted-angled TSAW (TaTSAW)-induced radiation force for particle focusing and particle separation. Reprinted with permission from reference [66]. SAW—surface acoustic wave.

Excited by the IDT, TSAW will generate pressure waves that propagate in a fluid domain, where acoustic streaming is created by viscous attenuation [58]. Franke et al. demonstrated a microfluidic device based on TSAW-induced acoustic streaming that separates HaCaT cells, MV3 melanoma cells, and fibroblasts from mice with high purity and viability [59]. With the careful alignment of trajectory, the cells were deflected to the collect channel when TSAW was triggered and moved toward the waste channel when TSAW was off. In the experiment, they applied square wave input signals and varied their frequency from 100 Hz to 2 kHz. The result showed that the sorting efficiency was 100% at lower frequencies. Similarly, Schmid et al. introduced a fluorescence-activated cell sorter (FACS) with the use of TSAW-induced acoustic streaming [60]. In this system, when TSAW was activated, the initial Poiseuille flow was modified by acoustic streaming-induced flow, which deflected the cells. Therefore, by tuning the frequency, the cells were separated into different outlets based on various levels of fluorescent signals (Figure 3a). TSAW-induced acoustic streaming has also been used for size-independent separation. Bourquin et al. applied TSAW-induced acoustic streaming to separate and enrich circulating cells infected by parasite from non-parasitized red blood cells (RBCs), enabling detection at low levels of infection on chips [61]. Since the density of infected RBCs is lower than the uninfected RBCs, the denser cells (uninfected cells) were accumulated at the center while the lighter cells (infected cells) moved to the periphery via acoustic streaming-induced drag force (Figure 3b).

Acoustic radiation force initiated by TSAW can also direct particles’ trajectory for particle separation, which dominates flow when k-factor is bigger than 1. As mentioned in the theory section, acoustic radiation force has a strong relationship with the size, density, and compressibility of particles. Thus, many acoustic microfluidic applications have emerged to separate particles based on their size, density, and compressibility [62]. Destgeer et al. demonstrated an acoustofluidic separator composed of a pair of slant interdigital transducers (SIDTs) to create TSAW to achieve the three-way size-selective separation for polystyrene particles (3, 4.2, and 5 μm) [63]. As shown in Figure 3c, the right IDT with frequency $\;f_{r}$-generated radiation force deflected the largest diameter particles to the left side, and the left IDT with frequency $f_{l}$-created radiation force drove medium-sized particles to the right side, leaving the smallest particles in the middle of the microchannel; thus, triple separation was realized. In a follow-up of this work, they developed a method for size-independent separation of polymethyl methacrylate (PMMA), elastic polystyrene (PS), and fused silica (FS) particles using acoustic radiation force [64]. They found that PS, PMMA, and FS particles have different peak acoustic radiation forces under different wavenumbers (k). As shown in Figure 3e, when k ≅ 1.29, PS particles experience a peak acoustic radiation force, while FS and PMMA particles are slightly influenced by the force. For k ≅ 1.69, PMMA particles are expected to experience a higher acoustic radiation force than PS and FS particles. They also calculated acoustic impedance ($Z_{p}$) of these particles by combining the particle density ($\rho_{p}$) and speed of longitudinal waves within the particle ($c_{l}$), which will significantly influence the deflection characteristics of particles when they are exposed to TSAW-based ARFs [64]. For FS particles, since they have higher acoustic impedance than PMMA and PS particles, they are hardly influenced by the acoustic radiation force. By tuning the TSAW frequencies (i.e., adjusting the k for the different particles), the radiation force acted on particles can be tuned to deflect particles at different levels.

Using the combined effect of acoustic streaming and acoustic radiation force induced by TSAW, Collins et al. reported a novel mechanism for size-selective particle manipulation [65]. They experimentally investigated the wavelength effect, applied power effect, and channel geometry effect on streaming patterns and particle concentration. With 6 µm wavelength and 10 µm channel height, particles as small as 500 and 300 nm were trapped since the radiation force overcame the acoustic streaming induced drag force, while 100 nm particles were not trapped because the acoustic streaming induced drag force is stronger than the acoustic radiation force (Figure 3d). This separation effect is similar to the oscillating bubble-induced microstreaming and secondary radiation force, but the mechanism is substantively different, where a high frequency is used to directly produce streaming and acoustic radiation force directly on the particles. Compared with acoustic bubble-based separation, this method avoids the issue of long-term stability of microbubbles, but the biocompatibility needs to be further investigated.

The location and angle of IDTs have also been studied for TSAW-based separation. Ahmed et al. exploited a tilted-angled TSAW (TaTSAW)-generated acoustic radiation force onto the 4.8 and 3.2 µm particles in continuous flow [66]. In their system, two tilted-angled IDTs can produce TaTSAWs that propagate at 30 degrees with reference to the fluid flow directions (Figure 3f). Therefore, the tiled-angled IDTs allow the acoustic radiation force and drag force to determine the directions of particles in the microchannel. By tuning the k factor of particles and changing the position of IDTs, the first IDT can focus the mixture of particles without sheath flow and the second IDT can separate particles by acoustic radiation force.

### 4.2. SSAW-Based Separation

SSAW technology, resulting from the combination of the two counter-propagating traveling surface acoustic waves, has been widely used for microfluidics separation. As shown in Figure 1b, when SSAWs propagate through the piezoelectric surface, the SSAW field has multiple pressure nodes and anti-pressure nodes at the surface of the substrate. The objects actuated by SSAW are expected to experience the primary acoustic radiation force in fluids, which will give rise to the displacement of the objects. As mentioned in the theory part, the acoustic contrast factor ϕ depends on the density and compressibility of the objects, and affects the objects’ movement toward the pressure nodes or antinodes. Generally, the acoustic contrast factor is positive for biological cells and solid particles, and negative for lipids and gas bubbles. Thus, in general, cells and solid particles will be pushed toward the pressure nodes, and lipids and gas bubbles will be directed to the pressure antinodes in a liquid medium [67]. Meanwhile, it is important to design a microchannel or microchamber with appropriate parameters to determine the position and the number of pressure antinodes and pressure nodes. For a standing acoustic wave, the distance between two nodes or two antinodes is equal to half of the wavelength. To optimize the efficiency of separation, the wavelength can be tuned to determine the position of the nodes and antinodes. In addition, for continuous separation, flow rate is also a key factor that can affect the performance of the separation by affecting the migration time for particles toward the pressure nodes or pressure antinodes [68].

Shi et al. introduced a SSAW-based method for continuously separating different-sized microparticles [69]. They used two parallel IDTs to generate one-dimensional SSAWs in the microchannel, in which the pressure nodes are positioned in the center of the channel. The ARF acted on the larger microbeads and pushed them toward the pressure nodes located in the center, while smaller microbeads experienced smaller ARF and moved slower than larger microbeads. Using SSAW-induced acoustic radiation force, particles of three different sizes (1, 5, and 10 µm) were also efficiently separated by properly controlling the flow rate and designing the position of pressure nodes and antinodes [70].

**Figure 4:**
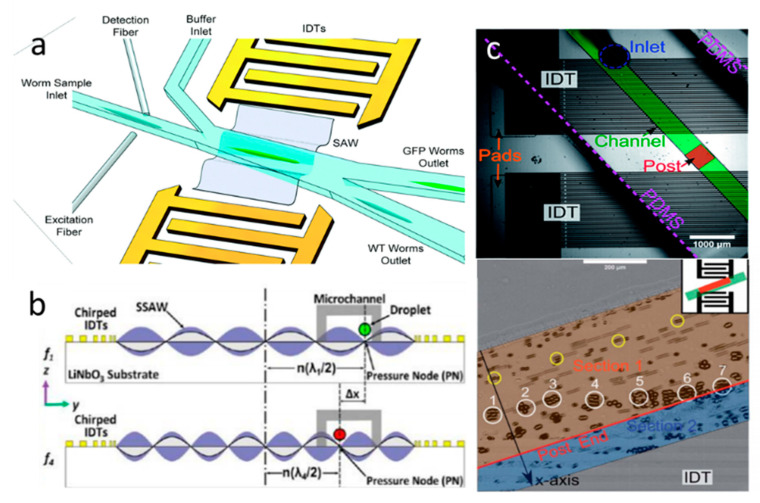
(**a**) Schematic illustration of an SSAW-based *C. elegans* sorting system. Reprinted with permission from reference [75]. (**b**) Cross-section view of the mechanism of SSAW-based droplet separation. By tuning the frequency, the location of the pressure node is changed. Reprinted with permission from reference [76]. (**c**) A size-based particle separation device that enables shaping SSAW by shifting the geometry of PDMS foil. Reprinted with permission from reference [81].

In addition to the abovementioned SSAW-based size-dependent separation, SSAWs are also able to separate particles based on other characteristics, such as density or compressibility. For instance, using SSAW, three types of uniform-sized cell-encapsulating beads have been separated based on density [71]. Since these beads have different acoustic factors (ϕ), which are determined by the density and compressibility of particles, they will experience different acoustic radiation forces. Therefore, driven by the lateral acoustic force, three types of beads showed different displacements toward the pressure nodes and, thus, were classified into three groups at the end of the microchannel. Recently, based on the different mechanical properties, Xie et al. have separated paraformaldehyde-treated and fresh HeLa cells with a recovery rate of 85% for the treated cells [72]. Fluorescent selective separation was also realized with the actuation of SSAW. Ren et al. presented high-performance microfluidics fluorescence-activated cell sorters (μFACS) by integrating SSAW and optical detection methods to separate individual cells [73]. When the fluorescent HeLa cell is detected in the separation region, the SSAW is turned on to deflect the particles to another outlet. Similarly, with the aid of a homemade laser-induced fluorescent system and SSAW, Nawaz et al. successfully sorted the fluorescent cells [74]. The green fluorescent protein (GFP)-expressing worms of different developmental stages have also been sorted by the fluorescence-based SSAW device with high throughput and accuracy (Figure 4a) [75].

As aforementioned, controlling the position of pressure nodes and antinodes can help to optimize separation efficiency. Guldiken et al. presented a microfluidic device for sheathless particle separation that includes two stages: the focus stage and the separation stage [68]. In the focus stage, the width of the microchannel is designed to produce only one node in the microchannel (equal to λ/2), thus, the particles are all centralized in the pressure node. In the separation stage, the microchannel is designed wider to form two nodes in the stream (equal to λ). Since the larger particles experienced stronger ARF, they are deflected toward the pressure nodes more quickly and isolated from the mixtures in a shorter SSAW exposure time. Additionally, compared to the design of the microchannel, tuning applied frequency is a simpler way to control the position of pressure nodes and antinodes. For example, Li et al. introduced a novel SSAW-based method that selectively separates water-in-oil droplets [76]. As shown in Figure 4b, the absolute distance between the pressure node and centerline (n(λ/2)) changes with tuning the frequency of the SSAW (f=c/λ, where c is the propagation velocity of the SSAW). As a result, excited by different frequencies, the droplets approached the different positions of pressure nodes and finally moved to the differing outlets.

The location and geometry of the transducer also play an important role in SSAW-based separation. Compared with standard IDTs, the focused interdigital transducers (FIDTs) can produce SAWs with high intensity and a narrow beam ratio [77]. Using a pair of FIDT-generated SSAWs, the separation performance has been improved with high-resolution and high-energy efficiency [78]. Similar to TSAWs, the impact position and location of the SSAWs to the separation efficiency have also been investigated by several researchers. Ding et al. developed an approach for separating MCF-7 cancer cells from the white blood cells using tilted-angled SSAWs (TaSSAWs) [79]. The two opposite TaSSAWs form a line of pressure nodes that is unparalleled with the microchannel. Comparing with the SSAW, the TaSSAW has the ability to separate the particles with a larger distance, enabling highly efficient and sensitive separation. Using TaSSAWs, Zhao et al. demonstrated a disposable device that can separate particles with a wide range from 200 nm to 10 µm [80]. Instead of changing the geometry of the IDT, Rambach et al. introduced a simple and flexible separation technique for different-sized particles which enables shaping of the SSAW by shifting the PDMS foil’s geometry [81]. By orienting the PDMS channel with a post diagonal to the IDTs, the acoustic force was localized at a specific area, in which 15 µm microbeads were deflected by acoustic radiation force and 10 µm microbeads still followed the direction of the fluid flow due to the stronger drag force (Figure 4c).

Parallel to experimental studies of SSAW-based separation, several numerical studies have been proposed for improving separation efficiency. Recently, Shamloo et al. presented a numerical method of SSAW-based separation of platelets, red blood cells, and white blood cells [82]. In this case, to find the maximum separation efficiency, they studied the influence of applied voltage to the performance of separation. The results show that by increasing applied voltage, cells experienced increasing acoustic radiation force and cell trajectories were more skewed to the center of the channel. Moreover, increasing the distance between the IDT’s edge and channel edge, cells were less forced to move to the centerline of the channel due to the decreased radiation force.

## 5. Exemplary Applications of Acoustic Micro-Object Separation

### 5.1. Cell Separation

Cell separation is a critical step in research and clinical testing. The acoustic cell separation method has been performed on a variety of biological samples for many applications, such as isolating blood cells, cancer cells, immune cells, sperm cells, etc.

Li et al. have successfully sorted white blood cells (WBCs) from lysed blood samples using an SSAW-based microfluidic device [83]. Due to the ARF generated by SSAW, the blood debris flowed with the stream through the lower outlet and WBCs were collected through the upper outlet with the purity up to 97%. Utilizing a similar mechanism, Dykes et al. have efficiently removed platelets from peripheral blood progenitor cell (PBPC) products in a microchannel [84]. They further demonstrated that the viability and progenitor cell colony-forming ability of collected PBPC products were preserved. With the use of an acoustic bubble-generated microstreaming, Phan et al. have focused RBCs into a defined range, which can be further applied in the separation process [85]. They found that the optimal focusing effect occurs when the bubble’s membrane is almost flat. Similarly, using microstreaming-induced drag force and secondary radiation force, Patel et al. separated the smaller RBCs from K562 cells actuated by an array of angled air–liquid membranes [20]. The results show that the viability of cells is achieved 94%–100%.

There are several numerical studies for blood cell separation, which not only reduce the expenses before the trials, but also help to avoid error in the experiments. As an example, Shamloo et al. demonstrated a numerical SSAW-based separation method of platelets, red blood cells (RBCs), and white blood cells (WBCs) by acoustic radiation force [82]. To improve the separation efficiency, the same group presented a simulation approach to separate WBCs from other blood cells in a trapezoidal channel instead of a conventional rectangular channel [86]. By optimizing the voltage and the trapezoidal leg angle, the distance from WBCs to the centerline achieved the minimum, and the more efficient separation were obtained. Gupta et al. proposed a simulation method for separating erythrocytes from blood plasma using a biofunctionalized layer in a SAW-based microfluidics platform [87]. Actuated by SAW, the erythrocytes were attracted at the nodal line located within biofunctional layers. This simulation result could be further validated in an experiment for detecting the infected erythrocytes, which also offers a guideline to develop a multifunctional microfluidics diagnostic tool.

**Figure 5:**
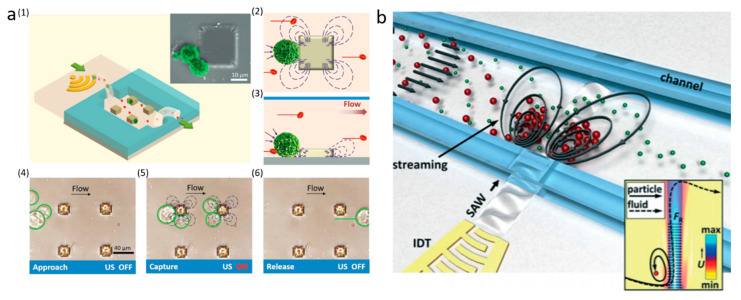
(**a**) Separation of CTCs from RBCs by acoustic radiation force and acoustic streaming. The actuated pillar array is able to trap the CTCs in a continuous flow. Reprinted with permission from reference [88]. (**b**) SAW-induced acoustic streaming separates human breast adenocarcinoma cells from RBCs. Reprinted with permission from reference [89].

Cancer cell separation has great benefits for cancer diagnosis and treament. Therefore, many acoustic cancer cell separation methods have been explored recently. A novel acoustic streaming-based isolation platform for separating cancer cells from the red blood cells is demonstrated by Lu et al. [88]. They utilized acoustic excited micropillars to generate the microstreaming, which can trap breast cancer cells, depending on their size (Figure 5a). They evaluated the trapping efficiency of three types of cancer cells (MCF-7 cells, SKBR-3 cells, and MDA-MB 231 cells) under different flow conditions. The results demonstrated that the separation efficiency can be 91% to 99% in both stop-flow and continuous flow. Similarly, using SAW-induced acoustic streaming, Collins et al. have separated human breast adenocarcinoma cells (MDA-231) from RBCs [89]. Since the drag force generated by acoustic streaming scales with the size of cells, the larger cancer cells were retained in the streaming vortices while the smaller sized RBCs were moved with the fluid flow, as shown in Figure 5b. Since cancer cells are rare in blood in the early stages, it is important to create a high-throughput microfluidic device for efficient separation. Recently, using a tilt-angled SSAW system, a high throughput CTC separation is achieved [90]. In this work, CTCs with low concentration have effectively separated from three patient samples with an 83% recovery rate.

In addition to separating cancer cells with different sizes, acoustic separation methods are also able to sort cancer cells of the same size, which is more challenging and needs to rely on properties other than size. Based on the biological characteristics, Faridi et al. proposed a size-independent acoustic separation method by utilizing antibody-functionalized microbubbles [91]. Actuated by a PZT transducer, antibody-functionalized polymer-shelled microbubbles selectively migrated the target cells (MD-Cell) from pressure nodes to pressure antinodes in a microchannel, and, thus, the selective separation of specific cancer cells was realized. Yang et al. applied ARF to separate viable and nonviable MCF-7 cells based on their different biological properties [92]. It provides an efficient and selective way to purify viable cells from the mixture of live and dead cells, showing great promise in disease treatment. Additionally, Karl et al. found that beyond a specific medium density, the viable and dead K562, MCF-7, and A498 cells can be separated using acoustic standing waves [93]. By measuring and analyzing the compressibility of cells, some studies proved that the compressibility of cancer cells is higher than their normal counterparts [94,95]. Therefore, acoustic cancer cell separation could benefit from the different compressibilities of cells.

With the advantages of being fast, convenient, and multifunctional, the integrated multi-stage microfluidic system holds great promise in simultaneous cell separation and analysis. By integrating TSAWs and SSAWs, CTCs and RBCs have been insolated in the bloodstream on a multi-stage microfluidics platform [96]. In this study, SSAWs were used to align CTCs and RBCs in a pressure node at the first part of the microfluidics device, while the unidirectional radiation force created by TSAWs enabled isolation of CTCs from RBCs. Antfolk et al. developed an integrated system for simultaneous size-based separating and concentrating two types of cancer cells (MCF7 and DU145) from WBCs [97]. From the results, the cancer cells can be isolated effectively with 88% efficiency. Moreover, A549 lung cancer cells, another type of cancer cells, have been isolated from the WBCs, relying on a novel three-step acoustic separation method with high efficiency (up to 92%) [98]. In this device, three zones with different applied frequencies are designed for alignment, separation, and trapping of CTCs, respectively.

Acoustic cell separation has been applied in forensic analysis. To obtain sexual assault evidence, an acoustic separation method for sperm cells from epithelial cell lysates is demonstrated [99]. Due to the different sizes, the sperm cells were trapped by the acoustic force while the epithelial cell lysates were not affected by the acoustic force, which allowed the separation of female DNA from male DNA in the next step. Most recently, Xu et al. demonstrated a similar method for the separation of sperm cells from female DNA using a three-layer (glass–PDMS–glass) microfluidic device [100]. The complete separation process takes only about 15 min, which saves significant time for analysis of suspects’ DNA compared with conventional separation methods.

Acoustic cell separation also has been applied in immunology. Hao et al. separated T cells from cell mixtures (Jurkat cells/K562 cells) on a shear horizontal surface acoustic wave (SHSAW) sensor-based microfluidic platform [101]. Lenshof et al. presented a novel acoustic separation method for the separation of CD4+ lymphocytes (a type of T cells that play an important role in the immune system) from PBPCs [102]. This acoustic-based separation method enables simultaneous bead-mediated multiple cell separation by using affinity beads with different acoustic properties, which is difficult to realize with current standard magnetic-based cell separation technology.

### 5.2. Protein Separation

**Figure 6:**
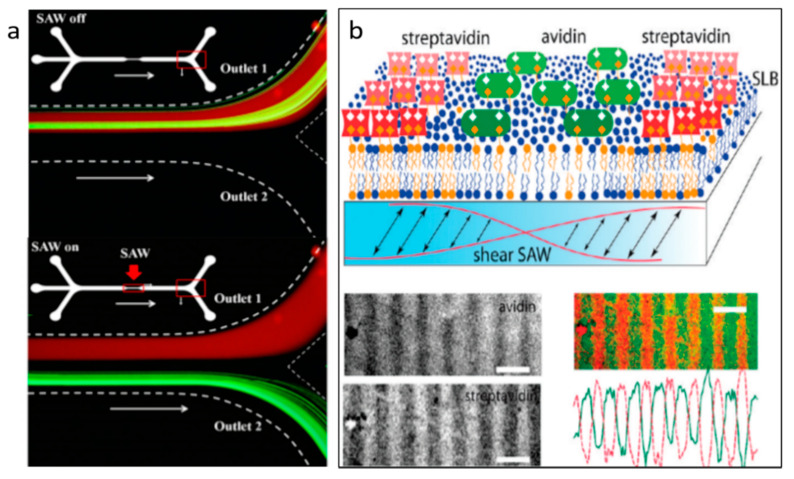
(**a**) When SAW is on, the green fluorescent particles attached to the thrombin are deflected from red fluorescent particles in continuous flow due to the acoustic radiation force. Reprinted with permission from reference [106]. (**b**) Streptavidin and avidin are separated on supported lipid bilayers by SSAW actuation. Reprinted with permission from reference [107]. SLB—supported lipid bilayers.

Protein plays an important role in metabolic and immune response, DNA replication, and molecule transportation [103,104,105]. By detecting and analyzing the target protein, many diseases can be diagnosed. Recently, acoustic separation has emerged as a great tool to selectively separate target proteins. By integrating high-frequency SAWs with a PDMS microchannel, Ahmed et al. developed a method for the separation of a target protein (thrombin and mCardinal2) from non-target biomolecules in a continuous flow [106]. In their system, streptavidin-functionalized PS particles are used to capture aptamer-labeled thrombin. Once the SAW is turned on, the thrombin-attached PS particles deflect from the mixtures due to the impact of ARF. They also demonstrated the separation of thrombin-attached green fluorescent PS particles from a red fluorescent protein, mCardinal2 (Figure 6a). Rather than separating protein in a continuous flow, Neumann et al. separated different proteins on a supported lipid bilayers (SLBs) platform, which is their natural environment [107]. Actuated by SSAWs, the streptavidin and avidin are separated toward pressure antinodes and nodes, respectively, due to their difference in densities (Figure 6b).

### 5.3. Virus Separation

**Figure 7:**
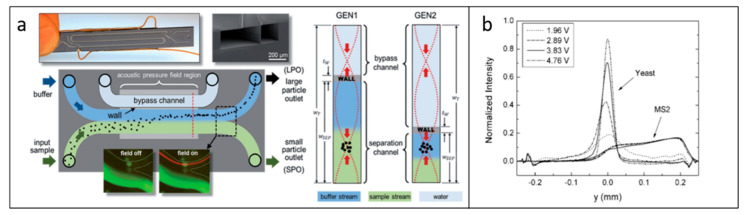
(**a**) Sorting of dengue virus particles and human Raji lymphocytes by acoustic radiation force. Reprinted with permission from reference [108]. (**b**) With increasing voltages, yeast were influenced by increased acoustic radiation force, while MS2 viruses were not influenced. Reprinted with permission from reference [109].

A virus is an infected biological agent that can spread disease to all life forms. Therefore, separation and detection of viruses are useful and necessary for viral disease diagnosis and treatment. Due to the advantage of being non-contact and label-free, acoustic separation techniques have been explored to separate viruses from other cells. As an example, a novel “transparent wall” approach based on PRF has been demonstrated to sort dengue virus particles (50 nm diameter) from human Raji lymphocytes (5–8 μm diameter) (Figure 7a) [108]. In this work, they controlled the location of the nodes and created asymmetric pressure nodes by designing a thin silicon wall to subdivide the microchannel into two parts, which is virtually transparent to ultrasound. Due to the PRFs, the larger human Raji lymphocytes were driven toward the asymmetric pressure nodes and separated from dengue viruses. This device is capable of achieving 70% purity for viral particle separation at 100 μL/min. This acoustic virus separation device not only demonstrates the capability of high throughput separation, but also enables reducing hands-on time for enriching virus samples for subsequent analysis steps. Relying on the similar PRF-based separation principle, Jung et al. have separated *Saccharomyces cerevisiae* (*S. cerevisiae*) from MS2 bacteriophage using an acoustic microfluidic particle filter [109]. The concentration profile for the MS2 virus kept constant since the acoustic radiation force did not affect the lateral displacement of the virus. However, the movement of yeast cells (*S. cerevisiae*) was influenced by the acoustic radiation force, as shown in Figure 7b. Eventually, 90% of sample purities were achieved by this separation method.

### 5.4. Bacteria Separation

**Figure 8:**
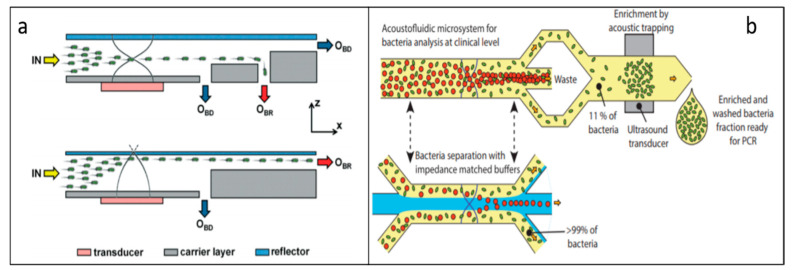
(**a**) Separating bacteria from water for water analysis using an optimized thin-reflected acoustic resonator. Reprinted with permission from reference [116]. (**b**) *E. coli* bacteria are separated from high concentrated blood samples and enriched for clinical sepsis diagnosis using polymerase chain reaction (PCR). Reprinted with permission from reference [115].

Since bacteria contamination has caused many health problems in recent years, effective isolation of bacteria becomes important in human infection, disease diagnosis, and food processing, as well as environmental analysis [110]. Recently, Ai et al. proposed an SSAW-based method to separate *E. coli* from peripheral blood mononuclear cells (PBMCs) with 95.65% purity [111]. Both *E. coli* and PBMCs moved toward the pressure node due to the positive acoustic contrast. However, *E. coli* and PBMCs are affected by different ARFs and are separated from each other due to the differences in size, density, and compressibility. Dow et al. isolated three pathogen species (*Pseudomonas aeruginosa*, *Staphylococcus aureus*, and *Escherichia coli*) from blood cells on a plastic acoustic-based microfluidic device [112]. The plastic microfluidic chips enable the development of a low-cost and disposable point-of-care system in future. Moreover, the plastic material has good compatibility with biological samples and is easy to fabricate [113]. According to the average of the results, RBCs were removed up to 85% and bacteria was retained from 45% to 60%. Similarly, using the Ta-SSAW technique, Li et al. demonstrated a size-based acoustic device to separate human blood and *E. coli* with 96% purity [114]. Later, using BAWs, 90% of bacteria has been separated from high-concentrated blood samples with a throughput of 400 µL/min [115]. After integrating with an acoustic trapping module, this device could detect *E. coli* for a clinical sepsis diagnosis with high throughput and a high recovery rate (Figure 8b). A low-cost and disposable plastic acoustic-separation device has been created for separating mixtures of *E. coli* and RBCs [113]. Using an inexpensive CNC micro-milling technique, the authors optimized the geometry and achieved 95% RBC-removal efficiency with an 82% reduction in the power requirements.

For water quality analysis, Carugo et al. developed an optimized thin-reflected acoustic resonator to collect high-concentrated bacteria under the action of acoustic radiation force (Figure 8a) [116]. Using this developed thin-reflected acoustic resonator, the bacteria concentration reached 60-fold. Separation of *E. coli* and sub-micrometer particles has also been achieved by a two-dimensional acoustic focusing method [117]. This method can isolate *E. coli* from 0.5 µm particles with purity above 90%, which could be further applied in environmental applications.

### 5.5. DNA/RNA Separation

DNA/RNA separation is a process of extraction of DNA/RNA from biological samples, which is essential for studying the causes of a disease and diagnosing genetic diseases. It is also important for forensic analyses and viruses and bacteria detection. The conventional and currently used DNA/RNA separation method is gel electrophoresis, which is an effective technique based on the size and charge of the DNA/RNA. However, this conventional method is relatively slow, usually taking one or two hours, and has low throughput, generally including manual handling steps [118]. Recently, acoustic microfluidic DNA/RNA separation was developed to circumvent these drawbacks. Iranmanesh et al. demonstrated a micro-vortexing method to achieve rapid cell lysis and DNA extraction actuated by an acoustic transducer [119]. In their work, human cancer cell A549 is completely lysed with an operating transducer for 25 s. Following the cell lysis, magnetic binding, and DNA quantification, the micro-vortexing method was used for DNA extraction.

Acoustic separation has also been applied in RNA separation. Recently, Taller et al. were able to separate miRNA from the exosome by utilizing SAWs for the study and diagnosis of pancreatic cancer [120]. Comparing the existing method with a 13 h processing time, this acoustic-based RNA separation method shows a significant efficiency improvement, taking only approximately 1.5 h to complete. By integrating a 50–250 nm tall nanoslit with acoustic waves, Miansari et al. proposed a pumpless size-exclusion separation method [121]. Acting by SAWs, only the particles that are smaller than the height of nanoslit can pass through. Since the size of DNA and RNA can be smaller than the height of nanoslit, it has great potential to be applied for DNA/RNA extraction.

## 6. Conclusions and Future Perspective

In this review, we reviewed the current state-of-the-art acoustic microfluidic separation techniques and categorized them as BAW-based separation and SAW-based separation depending on their work principles. By controlling the acoustic radiation force and drag force, these techniques are able to separate microparticles with different physical properties (size, density, and compressibility). The separation methods reported in this review have been applied for the separation of a wide range of biological samples, including blood cells, cancer cells, bacteria, viruses, DNA/RNA, and proteins, which has great potential to be used in biological research and clinical sciences.

Due to the advantages of being contactless, biocompatible, highly controllable, and versatile, acoustic microfluidic separation techniques continue to grow and expand in recent years. However, to realize their full potential, there are some limitations still required to be overcome in future. For example, the throughput of acoustic microfluidic separation devices is relatively low in a single-channel microfluidic device [122]. Moreover, acoustic separation microfluidic devices usually need to integrate with bulky instruments, such as power supply, voltage amplifiers, and syringe pumps, which increases the complexity of the system. In addition, acoustic microfluidic devices are typically fabricated by soft lithography and PDMS replication with high resolution. This technique, however, requires carrying out in a clean room and has complex fabrication steps. In future, to increase the throughput of the target sample, parallelized or multilayer acoustic microfluidic devices could be further explored. In addition, flexible, portable, and disposable acoustic separation devices could be helpful for enhancing usability. To shorten fabrication time and reduce cost, future development of acoustic microfluidic separation devices would welcome simple and low-cost fabrication techniques such as 3D printing [123]. Finally, acoustic separation devices can be further integrated with other on-chip functional units, such as pumps, mixers, and sensors, for more sophisticated multiple biomedical and clinical applications.
